# Tuning Anticancer Activity and Antimicrobial Response of ZnO Nanoparticles Through Halogenosilane Surface Modification

**DOI:** 10.3390/ijms27125388

**Published:** 2026-06-15

**Authors:** Mariana Bușilă, Aurel Tăbăcaru, Andreea Veronica Botezatu, Alina-Mihaela Ceoromila, Ana-Maria Moroșanu, Jeremias Muazeia, Jorge Humberto Gomes Leitão, António Pedro Matos, Fernanda Marques

**Affiliations:** 1 Department of Manufacturing Engineering, Faculty of Engineering, “Dunărea de Jos” University of Galați, 111 Domnească Street, 800201 Galați, Romania; mariana.busila@ugal.ro; 2 Department of Chemistry, Physics and Environment, Faculty of Sciences and Environment, “Dunărea de Jos” University of Galați, 111 Domnească Street, 800201 Galați, Romania; andreea.botezatu@ugal.ro; 3 Research and Development Center for Thermoset Matrix Composites, Cross-Border Faculty, “Dunărea de Jos” University of Galați, 111 Domneasca Street, 800201 Galați, Romania; alina.cantaragiu@ugal.ro; 4 Institute of Biology Bucharest, Romanian Academy, 296 Splaiul Independenței Street, 060031 Bucharest, Romania; anamaria.morosanu@ibiol.ro; 5 iBB—Institute for Bioengineering and Biosciences, Instituto Superior Técnico, Universidade de Lisboa, Av. Rovisco Pais, 1049-001 Lisboa, Portugal; jeremias.muazeia@tecnico.ulisboa.pt (J.M.); jorgeleitao@tecnico.ulisboa.pt (J.H.G.L.); 6 Associate Laboratory i4HB—Institute for Health and Bioeconomy, Instituto Superior Técnico, Universidade de Lisboa, Av. Rovisco Pais, 1049-001 Lisboa, Portugal; 7 Department of Bioengineering, Instituto Superior Técnico, Universidade de Lisboa, 1049-001 Lisboa, Portugal; 8 Centro de Investigação Interdisciplinar Egas Moniz, Campus Universitário Quinta da Granja, Monte Caparica, 2829-511 Caparica, Portugal; apamatos@gmail.com; 9 Centro de Ciências e Tecnologias Nucleares, Instituto Superior Técnico, Universidade de Lisboa, Estrada Nacional 10, Km 139.7, Bobadela, 2695-066 Loures, Portugal; 10 Departamento de Engenharia e Ciências Nucleares, Instituto Superior Técnico, Universidade de Lisboa, Estrada Nacional 10, Km 139.7, Bobadela, 2695-066 Loures, Portugal

**Keywords:** zinc oxide nanoparticles, surface modification, halogenosilanes, antitumor activity, antimicrobial response

## Abstract

Surface modification of zinc oxide nanoparticles (ZnO NPs) with organosilane capping agents represents an effective strategy to control their physicochemical and biological properties. In this work, we report for the first time the use of halogenosilanes, namely (3-chloropropyl)trimethoxysilane (CPTMS), (3-bromopropyl)trimethoxysilane (BPTMS) and (3-iodopropyl)trimethoxysilane (IPTMS), for the surface functionalization of ZnO NPs obtained by chemical precipitation. Structural and morphological characterization (PXRD, TEM, SEM-EDX and FTIR) confirmed successful surface modification and revealed a significant particle size reduction from ~31 nm for unmodified ZnO to ~8 nm for BPTMS-modified ZnO (ZnO_b). The biological evaluation showed that halogenosilane-modified ZnO NPs exhibit enhanced cytotoxic activity against prostate cancer cell lines (PC3 and 22Rv1), with ZnO_b displaying the highest activity, likely associated with improved cellular uptake and increased reactive oxygen species (ROS) generation. In contrast, antimicrobial assays revealed only moderate bactericidal effects against *Escherichia coli* and *Staphylococcus aureus* at relatively high concentrations (≥1250 µg mL^−1^), while no significant activity was observed against *Pseudomonas aeruginosa*, *Burkholderia contaminans* or *Candida* spp., within the tested range. These findings suggest that halogenosilane functionalization modulates the biological profile of ZnO nanoparticles by enhancing anticancer effects while also influencing microbiocidal activity, highlighting the role of surface chemistry in tuning biological selectivity. The present study supports the concept that rational surface engineering of ZnO-based nanoplatforms can be exploited to favor tumor-targeted activity over broad-spectrum antimicrobial effects, providing new perspectives for the design of application-oriented nanomaterials.

## 1. Introduction

The design of new inorganic nanomaterials with improved properties has started to take into account the surface modification through functionalization with organic capping agents. Such a strategy has become a central pursuit in nanomedicine, as it allows for combining therapeutic action with diagnostic or imaging capabilities [1]. Within this context, zinc oxide nanoparticles (ZnO NPs) represent a remarkable case of a single material spanning multiple research fields and scientific disciplines. Key features include wide bandgap, strong exciton binding energy and relatively simple preparation methods. Such properties render ZnO NPs highly attractive for applications ranging from electronics to catalysis and also for biomedical uses [2,3,4,5,6,7].

Pristine ZnO NPs are known for their tendency to agglomerate in aqueous media and release Zn^2+^ ions uncontrollably, thus raising concerns over reproducibility and safety [8]. Consequently, surface modification has become a useful strategy to modulate nanoparticle growth, aggregation behavior, surface reactivity and biological interactions. In suspension, however, colloidal stability must be evaluated through dedicated measurements such as zeta potential, hydrodynamic diameter and time-dependent dispersion stability. This is particularly relevant for ZnO nanoparticles, whose aggregation behavior and apparent hydrodynamic size are strongly affected by pH, time and medium composition [9]. In the realm of surface modification, the approach of organosilane functionalization stands out. In fact, silanes can form robust covalent bonds with hydroxylated ZnO surfaces, imparting stability and allowing fine-tuning of surface polarity [10,11]. In this context, silanes are not merely stabilizers but act as chemical tools to reshape ZnO’s physicochemical and biological behavior.

The biological performance of ZnO is strongly influenced by particle size and morphology. Smaller ZnO NPs generally exhibit higher surface reactivity, enhanced cellular uptake and stronger antimicrobial and anticancer activities. However, these intensified properties sometimes occur at the cost of elevated cytotoxicity toward healthy cells [12,13,14,15]. For instance, hepatocyte and mesenchymal stem cell studies have revealed clear size-dependent changes in oxidative stress and gene expression of genes related to senescence [16,17]. Morphological variations, such as nanosheets compared with flower-like structures, can also influence biological activity. For instance, sheet-like ZnO was shown to maintain dispersibility and superior antibacterial efficacy even under varying conditions [18].

The importance of defects has also been emphasized. Oxygen vacancies are known to facilitate ROS generation, even in the absence of light [19]. Intentional doping with gallium [20], magnesium [21] or silver [22] has been exploited to increase defect concentrations, thus amplifying cytotoxic and antibacterial effects. The performance of ZnO nanomaterials highlights the close interplay between size, shape and defect chemistry, which are intertwined with surface chemistry [23,24].

Organosilanes have become a central subject in tailoring ZnO for biomedical applications. For example, (3-glycidyloxypropyl)trimethoxysilane (GPTMS) coatings were shown to enhance colloidal stability while maintaining antibacterial activity against *E. coli* and *S. aureus* [25]. Increasing GPTMS content yielded smaller particle sizes, which promoted enhanced anticancer effects against ovarian cancer cells through ROS-mediated apoptosis and autophagy [24]. The influence of size in GPTMS-modified ZnO NPs has also been highlighted by increased antibacterial activity [26]. Moreover, GPTMS-modified ZnO retained antibacterial activity against multidrug-resistant *Enterobacteriaceae*, with improved stability during storage [27]. ZnO quantum dots capped with aminopropyltriethoxysilane (APTES) have shown promise in biosensing, bioimaging and antimicrobial activity, owing to their enhanced fluorescence and stability [28,29]. These examples support the *controlled surface–controlled performance* paradigm and show that organosilane functionalization can be an effective strategy for tuning ZnO NPs [30].

Yet, one avenue has remained largely unexplored, that is, the use of halogenosilanes. Conventional organosilane modifiers like GPTMS, APTES, or TEOS are typically selected to provide steric passivation, enhance water dispersibility, or deliver biocompatible organic groups. However, they generally exert passive roles regarding the intrinsic electronic properties of the core nanoparticle. In contrast, halogenosilane groups, such as chloropropyl, bromopropyl and iodopropyl, incorporate highly polarizable and electronegative terminal halogen atoms directly into the hybrid shell architecture. This structural variation offers a dual scientific advancement over traditional silane-functionalized ZnO systems. First, the distinct electronegativity and steric profiles of the halogens alter the hydrolysis-condensation kinetics at the ZnO interface during synthesis. This provides exceptionally tight control over nucleation. Consequently, it restricts secondary particle growth to a greater extent than standard alkyl- or epoxy-silanes, yielding ultra-small crystallites. Such a strategy effectively bridges a known synthesis challenge in controlling ZnO agglomeration. Second, the presence of localized halogen atoms significantly alters the surface energy, polarity and surface defect states of the nanoplatform. The chemical modification not only stabilizes the colloid but also actively dictates nanoparticle–cell interactions. This variation drives vastly superior cellular uptake and accelerates intracellular ROS generation in specific cancer cell lines. Such a particular pathway is well known to enhance preferential toxicity in malignant cells. Therefore, the presence of halogen atoms may influence the polarity, hydrophobicity and defect states, which could alter colloidal stability, ROS generation and interactions with cancer or microbial cell membranes. However, these effects remain hypothetical at the present stage and require dedicated mechanistic investigations for direct validation.

Halogenated silanes have already been employed in other material systems, often conferring higher reactivity or antimicrobial potential [31,32]. In biomedical surfaces, silane chemistry has also been extended to implantable biomaterials. Somasundaram reviewed how silanes, often in combination with metallic biomaterials, can improve bioactivity and antimicrobial resistance in implant coatings [33]. More recently, Morselli and co-workers demonstrated that sprayable PEG-silane/ZnO nanocomposite coatings can help prevent microbial colonization of titanium implants [34]. Complementary strategies further illustrate the breadth of ZnO nanomaterials. Halogenosilane green synthesis approaches have produced ZnO NPs with antibacterial activity and acceptable cytocompatibility [35]. In vitro studies have also highlighted the biomedical relevance of ZnO NPs by showing both antibacterial and cytotoxic effects. These findings emphasize the need for precise surface engineering to maximize biological benefit while minimizing risk [36].

However, to the best of our knowledge, no systematic comparative study has addressed surface modification of ZnO NPs with halogenosilanes containing chloropropyl-, bromopropyl- and iodopropyl-moieties, and evaluated how these halogenated surface groups affect both physicochemical features and biological responses. This knowledge gap motivated the present study. Herein, we report the functionalization of ZnO NPs utilizing chloropropyl-, bromopropyl- and iodopropyl-trimethoxysilane derivatives to evaluate how these specific surface groups modulate nanoparticle size, morphology, elemental composition, cytotoxicity, cellular Zn accumulation, ROS generation and microbiocidal activity. The ability of these ZnOs NPS to act as anticancer agents was evaluated in human prostate cancer (PC) cells, as PC remains one of the leading causes of death in men globally and the 5th leading cause of death worldwide [37]. Because PC3 cells are highly aggressive, androgen-independent human prostate cancer cells served as a primary model for testing [38,39]. The antimicrobial activity of ZnO NPs was also assessed towards the bacterial species *Staphylocccus aureus*, *Pseudomonas aeruginosa*, *Escherichia coli* and *Burkholderia contaminans*, and the fungal species *Candida albicans* and *Candida glabrata*. These microbial species were selected due to their ability to cause severe infections among human populations and their emergence as multidrug-resistant pathogens.

## 2. Results and Discussion

### 2.1. Synthesis and Morpho-Structural Characterization

The choice of chloro-, bromo- and iodopropyltrimethoxysilane was motivated by the progressive variation in physicochemical properties across the halogen series. Parameters such as atomic size, polarizability, electronegativity, steric hindrance and hydrophobicity may influence the organization and surface coverage of the silane layer, as well as nanoparticle growth and aggregation behavior during synthesis. Across the halogen series, changes in size and polarizability can alter the electrostatic and dispersion components of interfacial interactions [40], while halogen substituents may also participate in specific non-covalent interactions with electron-rich sites in biomolecules [41]. Therefore, after surface functionalization, these differences could affect nanoparticle–cell interactions, membrane affinity, surface polarity and oxidative stress-related processes.

The preparation of the new series of ZnO NPs modified with the halogenosilane species (3-chloropropyl)trimethoxysilane (CPTMS), (3-bromopropyl)trimethoxysilane (BPTMS) or (3-iodopropyl)trimethoxysilane (IPTMS) was performed by the in situ chemical precipitation method. The synthesis started from zinc(II) acetate dihydrate, fixed amounts of halogenosilanes corresponding to a 10% Si/Zn molar ratio, and potassium hydroxide, in ethanol under reflux for 3 h. The choice of using the 10% Si/Zn molar ratio was dictated by our previous observation that this was the maximum limit for reaching the highest surface coverage. Hence, the smallest ZnO NPs were formed upon surface modification with other organosilanes, such as (3-glycidyloxypropyl)trimethoxysilane (GPTMS) [24], (3-trimethoxysilyl)propylmethacrylate (MPS) [42], or vinyltrimethoxysilane (VTMS) [43,44]. Regarding biomedical applications, both the anticancer and antimicrobial properties of these nanoplatforms exhibit a clear size-dependency. For instance, our previous work showed that the smallest ZnO NPs, obtained via GPTMS-modification at the 10% Si/Zn ratio, displayed the optimal antibacterial performance against *E. coli* and *S. aureus* [24].

**Scheme 1:**
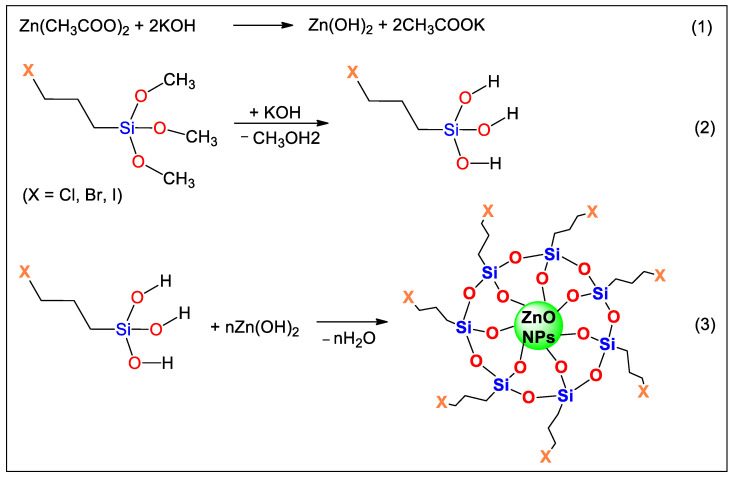
Possible mechanism for the formation of halogenosilane-modified ZnO NPs.

According to the proposed mechanism (Scheme 1), the formation of halogenosilane-modified ZnO NPs proceeds through three key steps: (1) zinc(II) acetate undergoes hydrolysis in the basic environment provided by potassium hydroxide to form zinc(II) hydroxide; (2) concomitantly, the halogenosilane molecules hydrolyze to generate the corresponding halogenosilanol species; (3) a self-condensation reaction occurs between the halogenosilanol species and zinc(II) hydroxide, driving the formation of ZnO particles coated with a silica network bearing the pendant halogenopropyl groups.

**Figure 1:**
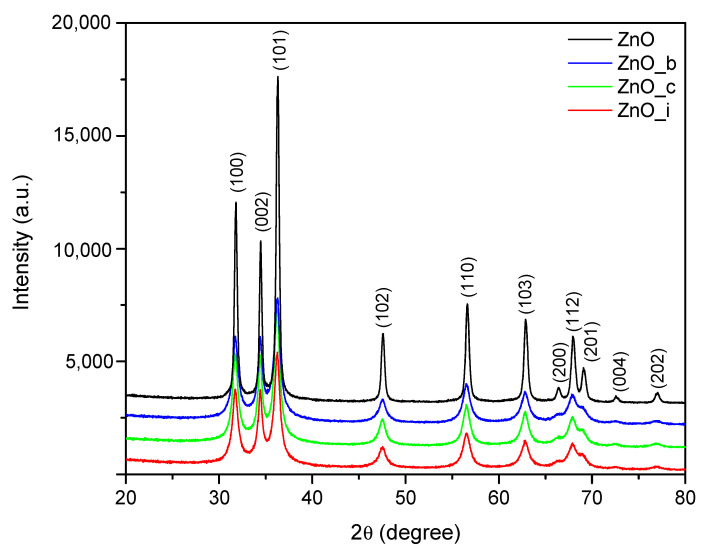
XRD patterns of unmodified ZnO NPs (black), chloropropylsilane-modified ZnO NPs (green), bromopropylsilane-modified ZnO NPs (blue) and iodopropylsilane-modified ZnO NPs (red).

X-ray diffraction (XRD) analysis allowed the confirmation of the presence of the zincite phase of ZnO in both unmodified and halogenosilane-modified samples, crystallizing in the hexagonal P63mc space group. The XRD patterns of all synthesized ZnO samples reveal the presence of the characteristic crystallographic planes (100), (002), (101), (102), (110), (103), (200), (112), (201), (004) and (202) in the 30–80° 2*θ* range (Figure 1). In the case of unmodified ZnO NPs, the XRD pattern shows well-resolved and more intense diffraction peaks. Upon ZnO surface coating with halogenosilanes, the diffraction peaks undergo a significant intensity decrease and broadening, as a consequence of crystallinity reduction, along with particle downsizing. As detailed in Table 1, surface functionalization leads to a noticeable reduction in crystallinity, dropping from 59.16% in unmodified ZnO NPs to 49.75% in the iodosilane-modified sample. Concomitantly, the average crystallite size decreases from ca. 21 nm in unmodified ZnO NPs to approximately 7 nm across all halogenosilane-modified samples. These trends highlight the highly efficient size reduction effect driven by these specific organosilane surfactants during the modification process. It is also worth mentioning that the observed differences in the crystallite size could also be due to eventual crystal microstructure changes induced by several factors, such as inhomogeneous strain, point defect, bending, dislocation generation and diffusion [45,46].

**Figure 2:**
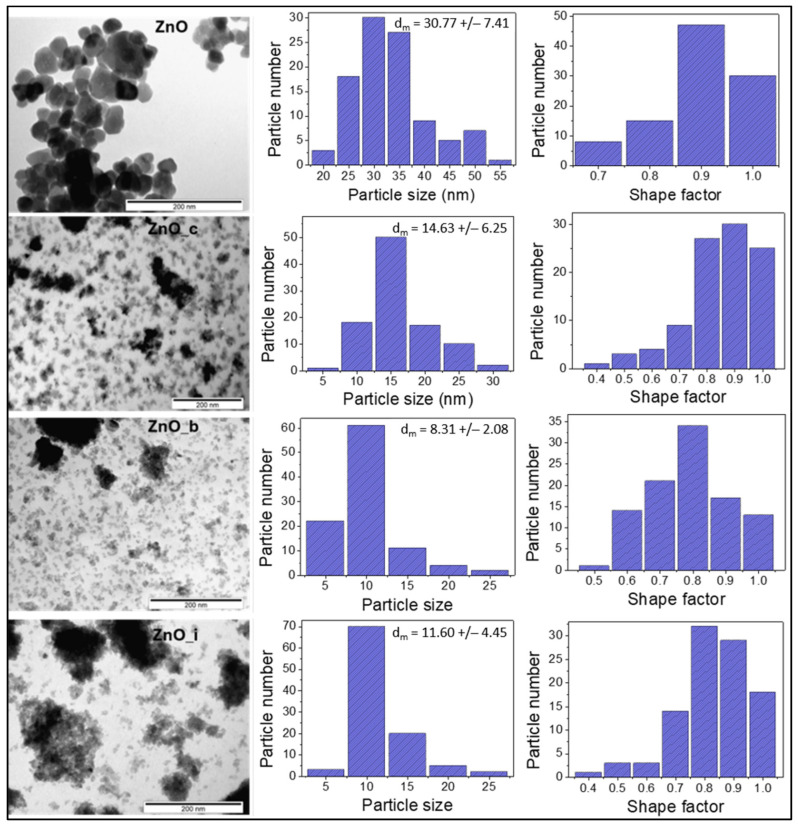
TEM micrographs (**left**), histograms (**middle**) and shape factors (**right**) for unmodified ZnO NPs, chloropropylsilane-modified ZnO NPs (ZnO_c), bromopropylsilane-modified ZnO NPs (ZnO_b) and iodopropylsilane-modified ZnO NPs (ZnO_i).

Transmission electron microscopy (TEM) allowed the clear influence of halogenosilane surface modification on the morphology and size of the synthesized ZnO NPs to be revealed. The unmodified ZnO NPs consisted of irregularly faceted and nearly spherical particles with a shape factor close to 1. These particles form dense agglomerates, with an average particle size of 30.77 ± 7.41 nm (Figure 2, sample ZnO). The corresponding size distribution was moderately broad, as indicated by a coefficient of variation (CV) of 24.07%, indicating extensive coalescence driven by high surface energy in the absence of stabilizing agents [47,48]. After modification with chloropropylsilane, the mean particle size decreased markedly to 14.63 ± 6.25 nm, and the particles appeared more discrete and uniformly dispersed. Their shape undergoes a slight distortion from the spherical one with a shape factor close to 0.9 (Figure 2, sample ZnO_c). However, the size distribution became more heterogeneous, as evidenced by a higher CV value (42.75%) and pronounced positive skewness (1.80), indicating the presence of a significant fraction of larger particles. This suggests that although nucleation is enhanced and growth is partially suppressed, the capping effect is not sufficiently uniform to fully control particle size dispersion. The interaction between chloropropyl groups and ZnO surface hydroxyls likely limits crystal growth, but does not completely prevent secondary aggregation processes [49,50]. The bromopropylsilane-modified ZnO NPs exhibited the smallest average size (8.31 ± 2.08 nm), along with a relatively low CV (24.97%) and near-symmetric distribution (skewness 0.25), indicating a more uniform and controlled nanoparticle population (Figure 2, sample ZnO_b). This indicates that bromopropysilane provided the most efficient capping among the three halogenosilane derivatives, combining adequate surface reactivity with enhanced steric hindrance. In contrast, the iodopropylsilane-modified ZnO NPs displayed a slightly larger mean diameter (11.60 ± 4.45 nm), with a relatively high CV (38.39%) and positive skewness (1.22), suggesting a broader and less uniform size distribution compared to ZnO_b (Figure 2, sample ZnO_i). This implies that the bulky iodine substituent reduced surface-binding strength and capping efficiency.

One-way ANOVA analysis revealed statistically significant differences in particle size among all samples (F = 336.47, *p* < 0.001). Tukey’s post hoc test confirmed that all pairwise comparisons were statistically significant (*p* < 0.05), demonstrating that the type of halogenosilane modifier exerts a strong and systematic influence on ZnO nanoparticle size and dispersion. Overall, the statistical analysis indicates that halogenosilane functionalization significantly reduces particle size, while its effect on size uniformity strongly depends on the nature of the halogen substituent. Among the investigated systems, bromopropylsilane yields the most controlled nanoparticle population, combining the smallest size with a relatively narrow and symmetric distribution. The descriptive statistics of the TEM-derived particle size distributions are summarized in Table 2.

Overall, halogenosilane modification significantly improved nanoparticle dispersion and reduced agglomeration, with the effectiveness of these modifiers following the trend ZnO_b > ZnO_c > ZnO_i > ZnO. The observed reduction in particle size and enhancement in morphological uniformity are consistent with earlier studies, which demonstrate that silane coupling agents can effectively passivate ZnO surfaces, disrupt agglomeration and limit the particle growth during chemical precipitation synthesis [51,52,53]. This finding supports the interpretation that the interplay between surface-bonding strength (Si–O–Zn formation) and steric effects of the halogen substituent governs growth inhibition efficiency. The smaller and more reactive halogens (Cl and Br) favor stronger surface anchoring, while bulkier iodine reduces capping uniformity, leading to partial coalescence. The observed differences among the three modified systems may reflect the combined influence of halogen size, polarizability, steric effects and silane-surface interactions on nanoparticle growth and surface organization. It should be noted that the improved dispersion observed by TEM reflects the morphological state of the dried nanoparticles under the preparation conditions used for microscopy. It should not be interpreted as direct evidence of colloidal stability in suspension. Dedicated measurements of zeta potential, hydrodynamic diameter and time-dependent dispersion stability would be required to assess the colloidal behavior of these samples in aqueous or biologically relevant media. Standardized approaches, such as OECD Test Guideline 318, further emphasize the importance of dispersion stability testing for evaluating nanomaterial behavior in test media [54].

It should also be noted that the XRD-derived crystallite size and the TEM-derived particle size describe different structural features. The crystallite size estimated from XRD peak broadening reflects the average dimension of coherently diffracting crystalline domains, whereas TEM provides a direct morphological estimate of the visible nanoparticles. Therefore, the two values are not expected to coincide in all cases. A larger TEM particle size compared with the XRD crystallite size may indicate that the observed particles contain several coherent crystalline domains or that small crystallites are assembled into larger particles or aggregates. In addition, lattice strain, point defects and surface disorder may contribute to diffraction peak broadening and lead to smaller apparent crystallite sizes. Thus, the differences observed between XRD and TEM, particularly for ZnO_c and ZnO_i, may arise from the combined effects of aggregation, partial polycrystallinity and strain/defect-related broadening. A more detailed separation of size and strain contributions would require dedicated analyses, such as Williamson–Hall or related line-broadening methods [55,56].

**Figure 3:**
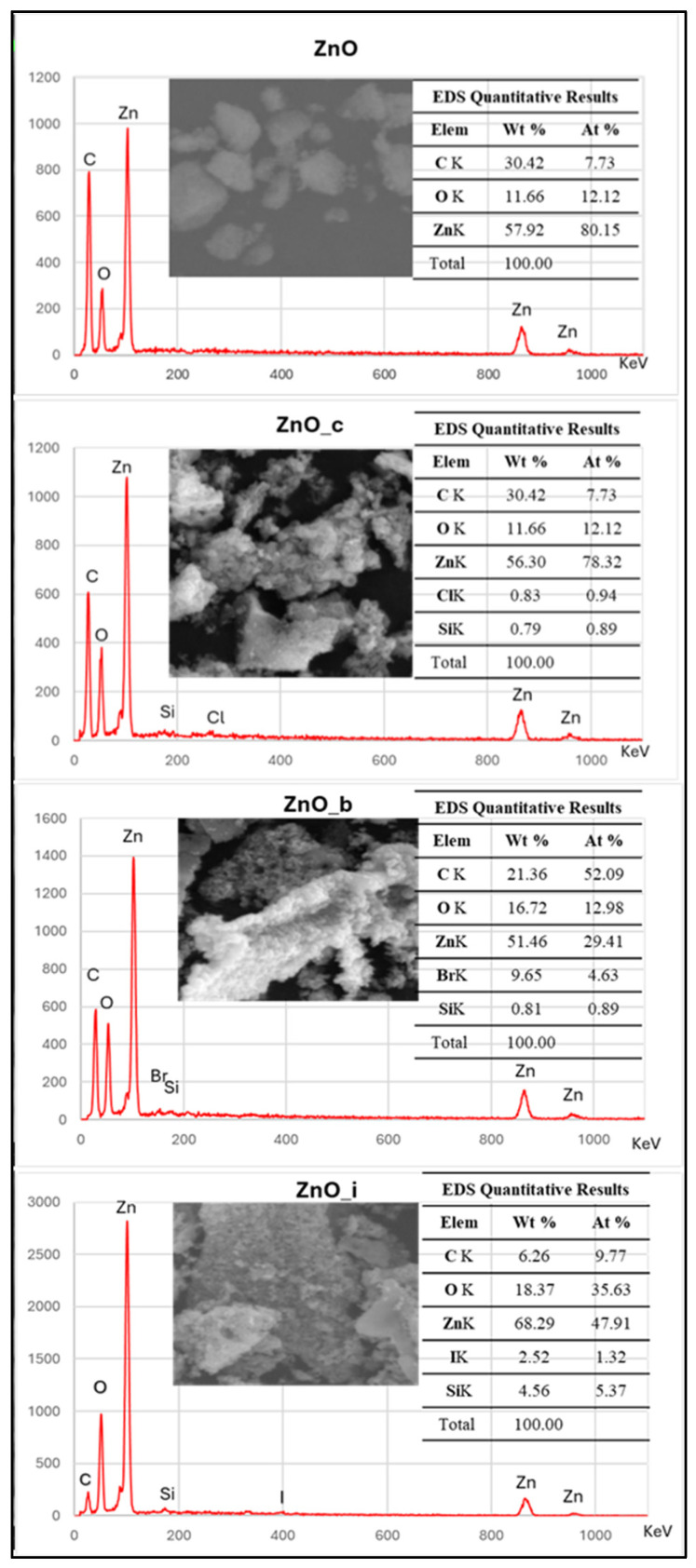
SEM-EDX analyses of unmodified and halogenosilane-modified ZnO NPs.

Quantitative EDX compositional analysis was conducted to further support successful halogenosilane modification of ZnO NPs (Figure 3). While the unmodified ZnO sample exhibits only Zn and O signals, upon modification, all functionalized samples show additional peaks corresponding to Si and the respective halogen (Cl, Br, I). The detection of Si in all modified samples verifies the presence of silane coupling on ZnO surfaces, while the simultaneous detection of halogens supports retention of the Si–propyl–X functionality post-modification. The decreasing Zn atomic fraction relative to the control reflects surface coverage by lighter elements (Si, C, halogen) and corroborates the formation of an organic–inorganic hybrid shell. Moreover, the relative atomic percentages suggest variable grafting efficiencies, governed by both the reactivity of the Si–O bond and the steric size of the halogen substituent.

**Figure 4:**
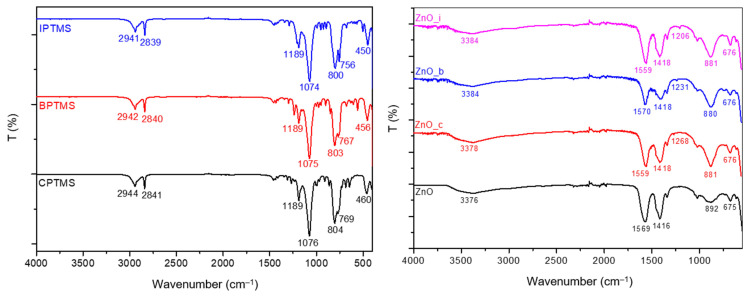
(**Left**): FTIR spectra of free halogenosilanes; (**right**): FTIR spectra of unmodified and halogenosilane-modified ZnO NPs.

Fourier transform infrared spectroscopy (FTIR) was employed to verify the success of halogenosilanes surface coating of ZnO NPs. The FTIR spectra of free halogenosilanes, as well as those of unmodified and halogenosilane-modified ZnO NPs, are given in Figure 4. In the FTIR spectra of free halogenosilanes, the strong absorption bands located at 1074–1076 cm^−1^ and 800–804 cm^−1^ were assigned to the stretching vibration of C–O bonds from methoxy groups and the symmetric stretching of Si–O–C bonds, respectively. In all halogenosilane-modified ZnO NPs, these bands completely disappear, as a consequence of the hydrolysis of methoxy groups bound to silicon. The new strong band observed at 880–881 cm^−1^ in all halogenosilane-modified ZnO NPs supports the formation of Zn–O–Si bonds and Si–O–Si bridges resulting from condensation processes involving hydrolyzed halogenosilane species and ZnO surface hydroxyl groups [29]. Together with the disappearance of the methoxy-related bands of the free silanes and the SEM-EDX detection of Si and the corresponding halogen atoms, these FTIR data are consistent with successful surface modification of ZnO NPs. However, FTIR alone cannot conclusively establish the detailed surface bonding environment or fully validate a core–shell structure. Therefore, the modified nanoparticles are more cautiously described here as surface-modified or core–shell-like ZnO systems. Additional surface-sensitive techniques, such as XPS or solid-state NMR, would be required to directly confirm the chemical environment of silicon and zinc at the interface and to validate the proposed surface architecture. Similar surface-coated or core–shell-like models have been proposed for silane- or silica-modified ZnO nanoparticles based on combined FTIR, XRD, TEM/SEM and EDX evidence [29,30,39,40]. However, in the absence of XPS or solid-state NMR, the present data should be regarded as supporting a surface-modified/core–shell-like model rather than conclusively proving a defined shell architecture.

The strong bands clearly observed at 756–769 cm^−1^ for the C–halogen bonds in the free halogenosilanes are no longer evident in the halogenosilane-coated ZnO NPs. They are likely to overlap with the strong bands already detected for the formation of Zn–O–Si bonds and Si–O–Si bridges. In the FTIR spectra of both unmodified and halogenosilane-modified ZnO NPs, the broad bands located in the range 3376–3384 cm^−1^ were assigned to the stretching vibration of hydroxyl groups of physisorbed water molecules. In the case of unmodified ZnO NPs, the weak broad band found at 892 cm^−1^ was assigned to the bending mode of carbon dioxide molecules, possibly physisorbed from air on the zinc (II) ions from the ZnO surface [57,58]. Also, in all ZnO samples, two strong bands observed at 1569 and 1416 cm^−1^ in unmodified ZnO NPs, 1559 and 1418 cm^−1^ in CPTMS-modified ZnO NPs, 1570 and 1418 cm^−1^ in BPTMS-modified ZnO NPs, and 1559 and 1418 cm^−1^ in IPTMS-modified ZnO NPs, were assigned to the asymmetric and symmetric stretching of remaining acetate groups from the initial zinc(II) precursor. After repeated washings, these groups could not be removed, thus giving rise to the consideration that the carboxylate groups were covalently attached to the zinc(II) ions [59].

The overall characterization supports the formation of smaller and less aggregated halogenosilane-modified ZnO nanoparticles in the solid state. However, their colloidal stability in suspension remains to be investigated in future studies using DLS, zeta potential and sedimentation or time-dependent dispersion assays.

### 2.2. Biological Properties

#### 2.2.1. Cytotoxic Activity

ZnO NPs have received much attention for their applications in cancer therapy. However, the underlying molecular mechanisms behind their anticancer activity remain unclear. It has been reported that ZnO NPs induce apoptosis mediated by reactive oxygen species generation [60]. The cytotoxicity of the halogenosilane derivatives has been reported to depend mostly on their physicochemical properties such as shape, size, and surface modifications. Size is, in fact, a very important factor, influencing the uptake and cytotoxicity [61]. Results of the cytotoxic effect of the unmodified and halogenosilane-modified ZnO NPs on prostate cancer cells PC3 (AR−, PSA−) and 22Rv1 (AR+, PSA+), normal prostate cells RWPE and fibroblasts HDF using the MTT colorimetric assay are presented in Table 3. After 24 h incubation with the cells, the halogenosilane-modified ZnO NPs showed a strong cytotoxic effect on both cancer cell types, and the unmodified ZnO showed lower cytotoxicity. In the normal prostate cells and fibroblasts, the ZnO NPs samples, in particular for the unmodified ZnO and ZnO_b, were slightly less active.

The higher cytotoxicity observed for the halogenosilane-modified ZnO NPs, particularly ZnO_b, should be interpreted with caution, as the present data do not allow the individual contribution of surface chemistry, particle size, aggregation state and intracellular zinc accumulation to be fully separated. The enhanced biological response may result from the combined influence of reduced particle size, improved dispersion, modified surface properties and increased interaction with cells.

Although the halogenosilane-modified ZnO NPs showed increased cytotoxicity toward both prostate cancer cell lines, the IC_50_ values obtained for RWPE normal prostate cells and HDFs were relatively close to those measured for cancer cells. This indicates that the therapeutic selectivity of the investigated nanoparticles remains limited under the present in vitro conditions. Therefore, the enhanced cytotoxicity observed for ZnO_b should be interpreted primarily as increased anticancer potency in prostate cancer models, rather than as evidence of strong cancer-cell selectivity. From a biosafety perspective, these results indicate that further surface optimization is required to widen the therapeutic window and reduce potential off-target toxicity toward non-malignant cells. Additional studies, including broader panels of normal cells, longer exposure times, hemocompatibility assays, 3D tumor models and in vivo evaluation, would be necessary to assess the translational potential of these nanomaterials.

#### 2.2.2. Cell-Associated Zn Accumulation Assessed by ICP-MS

**Figure 5:**
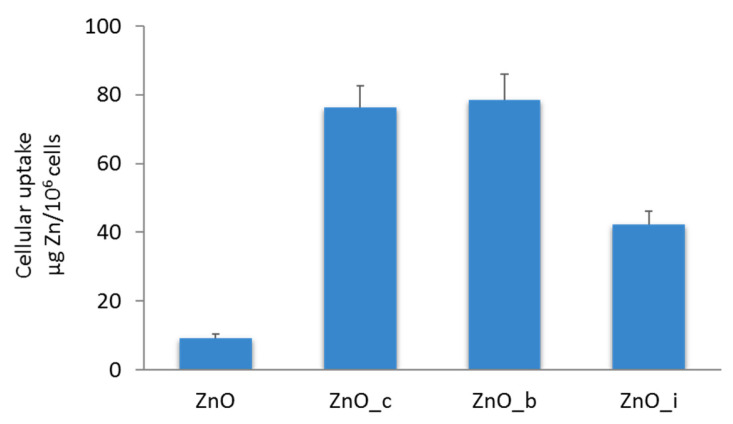
The uptake by PC3 cells, after exposure to unmodified ZnO, ZnO_c, ZnO_b and ZnO_i for 24 h at their IC_50_ values. The Zn levels were assessed by ICP-MS and expressed in µg Zn/10^6^ cells. The levels of Zn are indicated as mean ± SD.

The cellular journey of NPs into the cells begins by reaching the plasma membrane and entering the cell mainly through endocytosis [62]. ICP-MS was used to quantify total Zn associated with PC3 cells after 24 h exposure to ZnO and halogenosilane-modified ZnO NPs, followed by washing and acid digestion. The cellular uptake can be influenced by the size and the surface modification of the ZnO NPs. As can be observed in Figure 5, the cellular uptake in the PC3 cells (in terms of Zn levels) of unmodified ZnO was low compared to the halogenosilane-modified ZnO NPs, in particular ZnO_b and ZnO_c. These results are consistent with a stronger interaction and/or accumulation of the modified nanoparticles in PC3 cells.

However, these data should not be interpreted as direct evidence of a specific uptake mechanism. ICP-MS quantifies total Zn after digestion and does not distinguish between intact internalized nanoparticles, intracellular Zn^2+^ released from nanoparticle dissolution and strongly membrane-associated Zn-containing material. Although washing steps were used to reduce extracellular and loosely adsorbed particles, residual membrane-bound material cannot be completely excluded. Therefore, the ICP-MS results are more cautiously interpreted here as cell-associated Zn accumulation. Dedicated dissolution studies in the exposure medium, surface-stripping protocols, uptake inhibition assays or imaging-based localization studies would be required to distinguish among these possible contributions [63].

#### 2.2.3. Oxidative Stress by ROS

**Figure 6:**
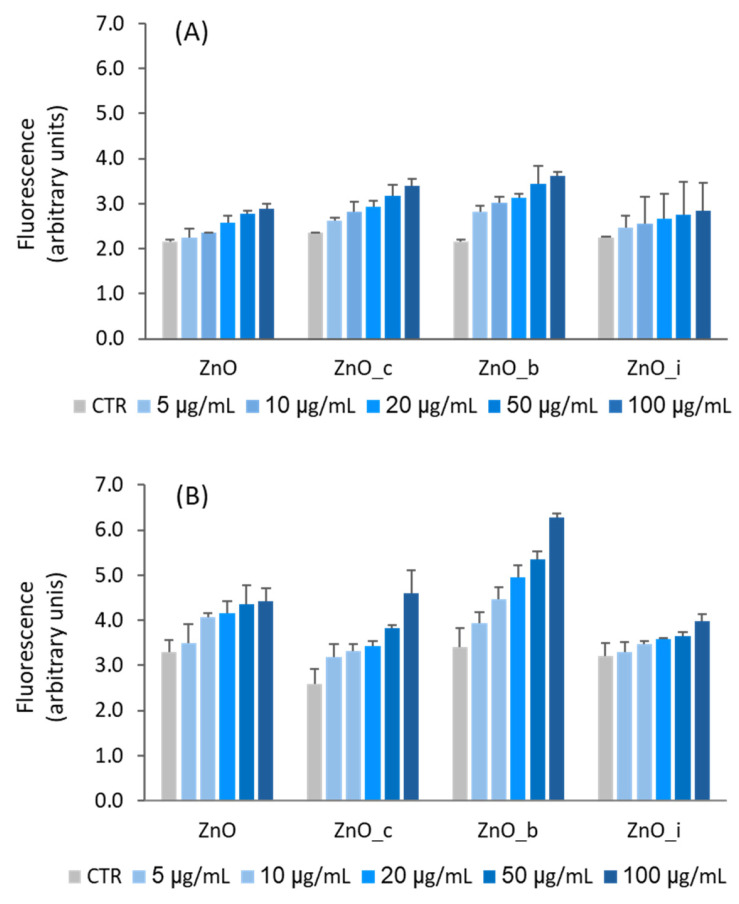
The ROS levels of PC3 cells after exposure for 24 h to different concentrations (5, 10, 20, 50 and 100 μg/mL) of the ZnO-NPs. The fluorescence signal of DCF was measured at 3 (**A**) and 24 h (**B**). Controls (CTR) represent untreated cells. Results (arbitrary units) are the mean ± SD from three independent experiments.

ZnO NPs have the unique ability to induce oxidative stress in cancer cells, which has been found to be one of the mechanisms of cytotoxicity [64]. Reactive oxygen species (ROS) generation and membrane lipid peroxidation were identified as a likely mechanism of ZnO NPs toxicity [24]. ROS generation was evaluated as a mechanistic indicator potentially associated with ZnO NP-induced cytotoxicity. The production of ROS (mainly peroxides) was measured using the cell-permeant indicator H_2_DCF-DA. The fluorescence signal of DCF was measured after 3 and 24 h. As can be observed from Figure 6, the production of ROS is time and concentration-dependent. After 24 h, an important increase in DCF fluorescence was observed in particular for ZnO_b. This trend is consistent with the higher cytotoxicity of ZnO_b in PC3 cells, suggesting that oxidative stress may contribute to cell damage.

However, the present data do not demonstrate a causal ROS-mediated cell death pathway. Confirmation of such a mechanism would require additional experiments using ROS scavengers or inhibitors, such as N-acetylcysteine, together with complementary analyses of downstream cell-death pathways. Therefore, ROS generation is interpreted here as an associated response rather than definitive proof of the primary mechanism of cytotoxicity.

It should be noted that the present cytotoxicity evaluation was restricted to prostate cancer cell models. PC3 and 22Rv1 cells were selected because they represent biologically distinct prostate cancer phenotypes, including androgen-independent and androgen receptor-positive/castration-resistant models, respectively. Therefore, the present data support the activity of halogenosilane-modified ZnO NPs in prostate cancer models, but they should not be generalized to other tumor types without further validation. Future studies including cancer cell lines from different tissue origins will be required to determine whether the observed cytotoxic behavior reflects a broader anticancer profile.

#### 2.2.4. Mechanisms of Cell Death

##### Apoptosis (Caspase 9 Activity)

The oxidative stress could lead to cell membrane damage and, depending on that, apoptosis may be triggered, which leads to cell death. Apoptosis is believed to be, for some nanoplatforms, the major mechanism of cell death in the cytotoxic response of ZnO NPs by the activation of caspases [65]. In this work, apoptosis was identified by measuring the level of caspase 9, the central initiator caspase.

**Figure 7:**
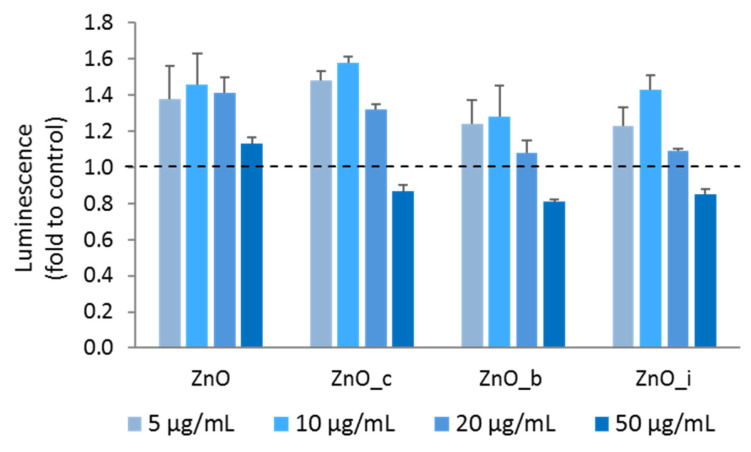
Apoptosis (caspase-9 activity) in PC3 cells. Results are the mean ± SD from three independent experiments.

Results in Figure 7 show that all the ZnO NPs induce caspase-9 activation to some extent, indicating that apoptosis-related processes may contribute to the cellular response. However, the caspase-9 profile did not directly parallel the cytotoxicity data. In particular, unmodified ZnO induced higher caspase-9 activation despite showing lower cytotoxicity than ZnO_b. This indicates that caspase-9-dependent apoptosis is not the only mechanism involved in the cytotoxic response. Also, the apoptosis levels of ZnO NPs do not correlate with the cellular uptake, as can be observed from Figure 5, which shows higher uptake for ZnO_b and ZnO_c. In the case of ZnO_b, the higher cytotoxicity together with comparatively lower caspase-9 activation and stronger ROS production suggests that additional caspase-independent or non-apoptotic mechanisms may contribute to cell damage. These may include necrosis-like processes, autophagy-related responses, ferroptosis or other oxidative stress-associated pathways. However, these mechanisms were not directly investigated in the present study and therefore remain to be clarified in future work using pathway-specific markers and inhibitors. Moreover, in the absence of ROS inhibition experiments, a causal role of ROS cannot be conclusively established.

The lower luminescence observed for ZnO_c, ZnO_b and ZnO_i could be attributed to the decreased number of cells, since the IC_50_ of these NPs is below 50 µg/mL.

These observations suggest that different mechanisms may contribute to cell death depending on nanoparticle surface properties, with unmodified ZnO favoring apoptosis-related pathways, while halogenosilane-modified systems, particularly ZnO_b, may involve additional ROS-driven or non-apoptotic mechanisms.

##### Morphological Features by SEM

**Figure 8:**
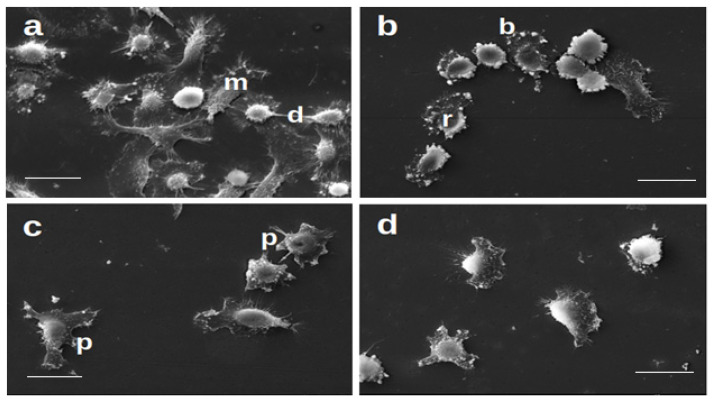
Scanning electron microscopy (SEM) of PC3 cells: non-treated cells (control) (**a**) and treated cells with ZnO (**b**), ZnO_b (**c**) and ZnO_c (**d**) at their IC_50_ values upon 24 h of treatment. Cell features: b = membrane blebs; d = dividing cells; m = migrating cells and p = membrane projections; bars: = 40 μm.

The morphological features of cells upon exposure to ZnO NPs were examined through the analysis of scanning electron microscopy (SEM). Cell-surface changes, including membrane blebbing and loss of cellular features, were observed (Figure 8). Figure 8 shows untreated cells (control (CTR), Figure 8a) presenting normal cellular morphology, including migrating cells (m) and dividing cells (d). In Figure 8b (ZnO), cells appear rounded (r) and are surrounded by membrane blebs (b), features compatible with apoptotic phenomena. In Figure 8c (ZnO_b), cells extend membrane projections (p), although often without the clear polarity observed in migrating control cells. Figure 8d (ZnO_c) shows membrane activity similar to that observed in Figure 8c. These observations suggest a marked contribution of apoptosis in ZnO-treated cells. In contrast, ZnO_b- and ZnO_c-treated cells still show signs of cellular activity, but these appear substantially altered and are probably dysfunctional when compared with CTR cells. Results are in agreement with those of caspase 9 activity assays (Figure 7) that show the ability of ZnO to activate caspase.

These morphological alterations are consistent with the biochemical assays and further support the involvement of oxidative stress and apoptosis-related processes. Moreover, the differences observed between ZnO and halogenosilane-modified samples reinforce the role of surface functionalization in modulating nanoparticle–cell interactions. However, SEM analysis alone cannot discriminate between apoptosis, necrosis, autophagy, ferroptosis or mixed cell-death mechanisms. Therefore, the SEM observations should be considered complementary evidence of altered cell morphology rather than definitive proof of a specific cell-death pathway.

#### 2.2.5. Antimicrobial Activity of ZnO and Halogenosilane-Modified ZnO Nanoparticles

The antimicrobial activities of ZnO and halogenosilane-modified ZnO NPs were assessed towards the Gram-negative *Pseudomonas aeruginosa*, *Escherichia coli*, and *Burkholderia contaminans*, the Gram-positive *Staphylococcus aureus*, and the fungi *Candida albicans* and *C. glabrata*. It should be emphasized that the values reported here correspond to minimal bactericidal or fungicidal concentrations (MBC/MFC), not minimal inhibitory concentrations (MIC). This distinction is important when comparing the present results with literature data, since MBC/MFC values reflect microbial killing, whereas MIC values reflect growth inhibition [66]. Therefore, MBC/MFC values are generally expected to be equal to or higher than MIC values, and direct comparison with MIC-based studies should be made with caution. Attempts to determine the MIC values based on the measurement of absorbance of microbial suspensions were hindered by the insoluble nature of the ZnO NPs. Therefore, we opted to incubate the microbial cultures in the presence of the ZnO and halogenosilane-modified ZnO NPs at final concentrations of 1000, 1250 and 1500 µg/mL for 24 h and enumerate the CFUs formed after 24 h of spreading 100 µL cultures on the surface of LB (bacteria) or PDA (fungi). The minimal concentrations of ZnO and halogenosilane-modified ZnO NPs, for which no microbial growth was registered, are shown in Table 4.

Concentrations up to 1500 µg/mL of the ZnO and halogenosilane-modified ZnO NPs were not bactericidal for *P. aeruginosa* and *B. contaminans*, and were also not fungicidal to *C. albicans* and *C. glabrata* (Table 3). Nevertheless, although the highest concentrations tested were not bactericidal against *P. aeruginosa* or *B. contaminans*, a concentration-dependent reduction in CFU counts was observed for these two bacterial species. For *E. coli*, the halogenosilane-modified ZnO NPs were more effective than the ZnO NPs. This was also the case for *S. aureus*, with the exception of ZnO-b, which presented a minimal bactericidal concentration identical to that of ZnO NPs.

ZnO nanoparticles’ antimicrobial activities have been tested by several authors. For instance, ZnO NPs MIC values of 62.5 µg/mL towards the Gram-negative *E. coli* and *Salmonella typimurium* were reported by Zhong et al. [67]. These authors reported MIC values of 31.25 µg/mL for the Gram-positive *B. subtilis* and *E. faecalis*. Interestingly, these authors also determined the percentage of viability of the same cultures and values of 5–10% viability were reported for ZnO NPs concentrations of 500 µg/mL. In our work, bactericidal activity (0% viability) was only achieved for concentrations of at least 1250 µg/mL (Table 3). Zhong et al. have not reported minimal bactericidal concentrations for the bacterial species tested.

Several mechanisms underlying the antimicrobial activity of ZnO nanoparticles have been described, including the generation of reactive oxygen species (ROS) in the presence of light. Additionally, nanoparticles can be adsorbed by the bacterial membrane, compromising its integrity and permeability, facilitating the internalization of nanoparticles that can lead to elevated and toxic Zn^2+^ concentrations within the bacterial cytoplasm [68]. In the present work, the mechanisms underlying the microbiocidal activity of the ZnO and halogenosilane-modified ZnO NPs were not addressed.

These results suggest that halogenosilane surface functionalization may influence the antimicrobial behavior of ZnO nanoparticles, particularly against *E. coli* and *S. aureus*, possibly through combined effects of particle size reduction and surface chemistry modification. This behavior, together with the enhanced anticancer activity, supports the idea that biological responses to ZnO nanomaterials can be modulated through rational surface engineering.

The relatively high bactericidal concentrations observed in this study may be related to several factors. First, the endpoint used here was complete killing, which is more stringent than growth inhibition. Second, nanoparticle aggregation in the microbial medium may have reduced the effective surface area and limited direct contact with microbial cells. Third, the halogenosilane surface layer, while beneficial for controlling particle growth and enhancing the anticancer response, may have partially modified the surface reactivity or Zn^2+^ release behavior of ZnO NPs, thereby reducing antimicrobial potency under the tested conditions. Finally, microbial susceptibility to ZnO-based nanomaterials is highly strain-dependent. Gram-negative bacteria such as *P. aeruginosa* and *Burkholderia* spp. possess complex cell-envelope structures and adaptive stress-response mechanisms that may reduce nanoparticle interaction, oxidative damage or metal-ion susceptibility. Since no dedicated antimicrobial mechanism or resistance assays were performed, these factors should be regarded as plausible explanations rather than experimentally demonstrated mechanisms in the present study. Future studies aimed at improving the antibacterial performance of these systems may explore mixed-metal oxide or hybrid nanostructures. For example, Cu-ZnO embedded in a polydopamine shell was recently reported as an antibacterial coating strategy, suggesting that combining ZnO with copper-based components may enhance antimicrobial activity through complementary mechanisms [69].

## 3. Materials and Methods

### 3.1. Reagents

Zinc acetate dihydrate (purity > 98%), potassium hydroxide (purity ≥ 85%), ethanol (purity = 98%), (3-chloropropyl)trimethoxysilane (purity ≥ 97%), (3-bromopropyl)trimethoxysilane (purity ≥ 97%) and (3-iodopropyl)trimethoxysilane (purity ≥ 97%) were purchased from Merck (Darmstadt, Germany) and used as received.

### 3.2. Synthesis of Halogenosilane-Modified ZnO NPs

The synthesis of both unmodified and halogenosilane-modified zinc oxide nanoparticles (ZnO NPs) was conducted via a controlled in situ chemical precipitation method. To ensure maximum surface coverage and minimize particle agglomeration, a fixed 10% Si/Zn molar ratio was maintained for all functionalized batches. Here, the Si/Zn ratio represents the molar proportion between the silicon atoms introduced via the organosilane capping agent and the zinc ions delivered by the metal precursor.

In a typical procedure, a standard batch was calculated based on 2.45 g of zinc acetate dihydrate (Zn(CH_3_COO)_2_·2H_2_O, 11.16 mmol) as the ZnO precursor. Based on this fixed amount of zinc, the corresponding mass of the respective halogenosilane modifier needed to achieve the 10% Si/Zn molar ratio (1.116 mmol) was calculated as follows: 0.222 g of (3-chloropropyl)trimethoxysilane (CPTMS), 0.272 g of (3-bromopropyl)trimethoxysilane (BPTMS), or 0.324 g of (3-iodopropyl)trimethoxysilane (IPTMS). To initiate the reaction, 10 mL of absolute ethanol was first introduced into a 50 mL round-bottom flask and brought to a stable temperature of 45–50 °C under continuous magnetic stirring. The pre-calculated mass of the specific halogenosilane (CPTMS, BPTMS, or IPTMS) was then added directly to the warm solvent. The temperature of this mixture was progressively elevated to 60 °C and held constant for exactly 10 min. This separate thermal step is essential to permit partial hydrolysis and chemical activation of the silane methoxy groups prior to core growth. After this activation window, 2.45 g of zinc acetate dihydrate was added directly to the solution under vigorous stirring. Concomitantly, a separate precipitating solution was prepared by dissolving 1.25 g of potassium hydroxide (KOH) in 5 mL of absolute ethanol at room temperature. This alkaline solution was then added dropwise into the main flask containing the zinc acetate and activated silane matrix. The dropwise addition rate was strictly maintained to ensure uniform nucleation boundaries and suppress uncontrolled crystal coalescence. Upon complete addition of KOH, the reaction assembly was brought to reflux and left under constant magnetic stirring for 3 h to facilitate complete core growth and covalent surface cross-linking. The resulting hybrid nanoparticles were isolated by centrifugation (9000 rpm for 5 min at 10 °C) and washed sequentially with three independent 5 mL portions of absolute ethanol. The isolated white powders were finally dried in a conventional oven at 100 °C for 2 h. ZnO NPs samples modified with CPTMS, BPTMS and IPTMS are denoted as ZnO_c, ZnO_b and ZnO_i, respectively.

For the preparation of the bare, unmodified control sample (designated simply as ZnO), the chemical precipitation was carried out using an identical procedure. Briefly, 2.45 g (11.16 mmol) of zinc acetate dihydrate was dissolved directly in 10 mL of absolute ethanol under constant stirring at 60 °C. Concomitantly, the precipitating solution of 1.25 g of KOH in 5 mL of ethanol was added dropwise to induce nucleation. All subsequent aging, reflux (3 h), centrifugation (9000 rpm), ethanol washing, and oven-drying (100 °C for 2 h) protocols were strictly maintained identically to those utilized for the halogenosilane-functionalized series to ensure a reliable baseline for morpho-structural and biological comparisons.

### 3.3. Nanoparticles Characterization

#### 3.3.1. XRD Analysis

X-ray diffraction (XRD) patterns were recorded on ground powders with an Empyrean (PANalytical, Almelo, The Netherlands) diffractometer, using Cu K_α1_ (λ = 1.540598 Å) radiation, equipped with a 2xGe (220) hybrid monochromator for Cu and a PIXcel3D detector. The analyses were performed using Bragg–Brentano geometry (“theta-2theta”) for angles between 10 and 80° 2θ degrees, with a scan step increment of 0.04° and an acquisition time per step size of 3 s. Sample preparation: a small amount of each sample was mounted on a single crystal silicon holder, which does not give diffraction interferences in the analysis range for the radiation emitted by a Cu anode. The sample was gently pressed on the holder so that the analyzed area was flat. For the identification of cell parameters and mean crystallite size, Rietveld refinement was performed in PANalytical High Score Plus v3.0e software. The agreement indices considered for the refinement are R_expected_, R_profile_, Weighted R profile and Goodness of fit.

The average crystallite size (*L*) of synthesized ZnO NPs was estimated by applying Scherrer’s Equation (1):

(1) $$B\left(2\theta \right)=\frac{K\cdot \lambda }{L\cdot \cos\theta }$$

where *B* is the full width at half maximum intensity (FWHM), *λ* is the X-ray wavelength, *θ* is the diffraction angle, and *K* is the Scherrer’s constant, whose value for spherical particles is 0.89 [70].

#### 3.3.2. TEM Analysis

Transmission electron microscopy (TEM) was conducted to analyze the morphology and size distribution of both unmodified and halogenosilane-modified ZnO NPs. The samples were examined using a JEOL JEM-1400 transmission electron microscope (JEOL, Toyko, Japan) operated at 80 kV accelerating voltage, equipped with a Quemesa CCD camera (Olympus Soft Imaging Solutions GmbH, Münster, Germany). For statistical analysis, at least 100 nanoparticles were measured per sample. Using the ImageJ software (version 1.54p, National Institutes of Health, Bethesda, MD, USA) (https://imagej.nih.gov/ij/, accessed on 4 April 2025), the crystal outlines were digitized and their dimensions measured. The resulting data were afterward used to generate histograms in OriginPro 8.5 software (OriginLab Corporation, Northampton, MA, USA), illustrating the average size of the nanoparticles. For each sample, a 1 mg/mL dilution in absolute ethanol was prepared and thoroughly dispersed using a Sonorex Digital 10 P ultrasonic bath (Bandelin electronic, Heinrichstraße, Berlin, Germany). Subsequently, working in duplicate, a droplet of the nanoparticle suspension was applied onto 200 mesh formvar-coated grids and allowed to air-dry.

Statistical differences between the particle size distributions of the investigated samples were evaluated using one-way analysis of variance (ANOVA) implemented in the OriginPro 8.5 software (OriginLab Corporation, Northampton, MA, USA). Post hoc multiple comparisons were performed using Tukey’s test to identify significant differences between individual sample pairs. The main statistical descriptors, including the mean particle size (average diameter), standard deviation (SD), minimum and maximum particle sizes, and mode, were calculated for each sample. The coefficient of variation (CV) was determined as the ratio between the standard deviation and the mean particle size, providing a normalized measure of dispersion. In addition, the polydispersity index (PDI) was estimated using the relation $PDI={\left(\sigma /\bar{d}\right)}^{2}$, allowing the evaluation of size uniformity across the nanoparticle populations. The asymmetry of the particle size distributions was qualitatively assessed through skewness analysis, based on the shape of the histograms.

#### 3.3.3. SEM-EDX Analysis

The morphology and chemical composition of ZnO samples were analyzed through scanning electron microscopy (SEM) using the FEI Quanta 200 system (FEI Company, Hillsboro, OR, USA) operating at 15 kV, coupled with an energy-dispersive X-ray spectroscopy (EDX) system. Specimens were prepared by dispersing the samples by sonication in ethanol and by depositing a few drops of the suspensions on carbon-coated grids. To minimize charging effects during SEM examination, the samples were coated with a thin gold layer deposited by sputtering using an SPI-Module system (SPI Module™ Supplies, West Chester, PA, USA).

#### 3.3.4. FTIR Analysis

Fourier transform infrared spectra (FTIR) were collected using a Nicolet iS50 FTIR spectrometer (Thermo Scientific, Waltham, MA, USA) equipped with a built-in ATR accessory, DTGS detector and KBr beam splitter. A total of 32 scans were added in the range 4000–400 cm^−1^, with a resolution of 4 cm^−1^.

### 3.4. Biological Assays

#### 3.4.1. Anticancer Activity

PC3 (PSA−, AR−) (ATCC, CRL-1435) and 22Rv1 (PSA+, AR+) (ATCC, CRL-2505) human prostate cancer cells were cultured in RPMI medium supplemented with 10% fetal bovine serum (FBS) (Gibco, Thermo Fisher Scientific, Waltham, MA, USA), RWPE-1 normal human prostate cells (ATCC, CRL-3607) were cultured in keratinocyte serum-free medium (K-SFM) supplemented with bovine pituitary extract (BPE) (0.05 mg/mL), epidermal growth factor (EGF) (5 ng/mL) and 10% FBS, and the human dermal fibroblasts (HDF) (Sigma-Aldrich, Merck, Darmstadt, Germany) were cultured in fibroblast growth medium (Sigma-Aldrich). Cultures were maintained in an incubator (Heraeus, Hanau, Germany) with a humidified atmosphere at 37 °C and 5% CO_2_. The cytotoxic activity of the halogenosilanes-modified ZNO NPs was evaluated by the MTT colorimetric assay as previously described [24]. For the assays, cells (1–2 × 10^4^ cells/200 µL) were suspended in culture medium, seeded into 96-well plates and incubated for 24 h. A stock suspension of each nanoplatform was prepared in PBS at a concentration of 1 mg/mL and sonicated for 5 min. Then, working suspensions of 1, 5, 10, 20, 50, 100 and 200 μg/mL were prepared in complete media and added to the cells. Untreated cells were also included as the negative controls. After treatment with the NPs, the cell culture medium was replaced with 200 μL of MTT solution in PBS (0.5 mg/mL), followed by 3 h of incubation at 37 °C. The resulting formazan product was solubilized with 200 μL DMSO, and the absorbance was measured at 575 nm. Each experiment was repeated at least twice, and each concentration was tested with at least six replicates. The IC_50_ values were calculated from dose–response curves using the GraphPad Prism software (vs. 6.0).

#### 3.4.2. Analysis of Cellular Zinc Content by ICP-MS

PC3 cells (~10^6^ cells) were seeded into T25 cell culture flasks with RPMI medium and incubated for 24 h. Afterward, the ZnO-NPs were added to the cells at their IC_50_ (µg/mL) in medium and incubated for 24 h at 37 °C. After treatment, the cell pellets obtained by centrifugation were washed with PBS and then were digested in 100 µL of HNO_3_ combining ultrasound (60 min at 60 °C) and microwave (350 W, 15 s) radiation for Zn quantitation by a Thermo X-Series Quadrupole ICP-MS Thermo Scientific, Rockford, IL, USA, as previously described [71].

#### 3.4.3. ROS Production

The production of ROS (mainly peroxides) was measured using the fluorescent probe H_2_DCF-DA (2′,7′-dichlorodihydrofluorescein diacetate) as previously described [72]. Briefly, PC3 cells (~2 × 10^4^/200 µL) in RPMI medium with 10% FBS (Gibco) were seeded in 96-well plates and left to adhere overnight. Then, the medium was replaced with the ZnO-NPs suspensions in complete RPMI medium at 5, 10, 20, 50 and 100 µg/mL and left for 24 h incubation. Afterward, the medium was replaced by a solution of 10 μM H_2_DCF-DA in colorless FluoroBrite™ DMEM (Gibco, Thermo Fisher Scientific, Waltham, MA, USA), and cells were incubated for 30 min at 37 °C. The probe solution was removed and substituted with the colorless medium. Dichlorofluorescein (DCF) fluorescence was measured at 492 nm excitation and 517 nm emission over time up to 24 h using a Varioskan LUX scanning multimode reader (Thermo Fisher Scientific). Results (mean ± SD) are expressed as arbitrary fluorescence units. The intensity of the resulting fluorescence is directly proportional to the amount of ROS present in the cells.

#### 3.4.4. Apoptosis Assay

The mechanism of cell death by apoptosis was evaluated by a Caspase-Glo^®^ 9 luminescent assay following the protocol of the supplier (Promega, Madison, WI, USA). The assay provides a luminogenic caspase-9 substrate in a buffer system optimized for caspase activity, luciferase activity and cell lysis. The assays were carried out in white-walled 96-well plates, with PC3 cells (~2 × 10^4^/200 µL) treated with the ZnO-NPs suspensions in medium at 5, 10, 20, and 50 µg/mL. After 24 h incubation, 100 μL of medium was removed from each well. Caspase 9 reagent was added in a 1:1 ratio, and the plate was shaken in an orbital shaker for 30 s at 300 rpm. The plate was incubated at room temperature for 1.5 h, protected from light. The luminescent signal produced by the luciferase reaction was measured using a Varioskan LUX scanning multimode reader (Thermo Fisher Scientific). The signal generated is proportional to the amount of caspase activity present.

#### 3.4.5. SEM Studies with Cells

PC3 cells were seeded over glass lamellae in 6-well plates at a density of 2 × 10^5^ cells in 2 mL cell medium and allowed to adhere for 24 h. Afterward, the ZnOs were added to the cells in medium at a concentration corresponding to their IC_50_ values, 66, 20 and 34 μM, respectively, for ZnO, ZnO_b and ZnO_c. After 24 h incubation, the medium was discarded, and the cell-covered glass lamellae were treated following a previously described method [73]. The samples were observed and photographed using a JEOL 5400 scanning electron microscope.

#### 3.4.6. Antimicrobial Activities of ZnO and Halogenosilane ZnO NPs

The antimicrobial activity of the ZnO, ZnO_c, ZnO_b and ZnO_i nanoparticles was assessed towards the Gram-negative *Pseudomonas aeruginosa*, *Escherichia coli* and *Burkholderia contaminans*, the Gram-positive *Staphylococcus aureus* and the fungi *Candida albicans* and *Candida glabrata*. Due to difficulties in determining MIC values based on spectrophotometric methods, the antimicrobial activities of the ZnO, ZnO_c, ZnO_b and ZnO_i nanoparticles were assessed by determining the minimal bactericidal or fungicidal concentrations [74,75]. For this purpose, masses of nanoparticles ranging from 1.506 to 2.250 mg were weighed and poured into the wells of 6-well polystyrene plates. Overnight liquid cultures of bacteria and fungi were carried out by loop inoculating 25 mL of Mueller-Hinton broth (MHB) and Yeast Potato Dextrose (YPD), followed by incubation at 37 °C with orbital agitation (250 rpm). Overnight grown cultures were diluted 1:100 in MHB (bacteria) or Roswell Park Memorial Institute (RPMI) (fungi) and grown for an additional 3 h at 37 °C with orbital agitation (250 rpm). Cultures were then adequately diluted in fresh media to obtain 5 × 10^5^ colony-forming units (CFU) per mL. Adequate volumes of freshly inoculated MHB or RPMI broth were added to the polystyrene plate wells to obtain nanoparticles final concentrations of 1000, 1250 or 1500 µg/mL. Plates were covered with aluminum foil to prevent light exposure and incubated for 24 h at 37 °C with agitation. After incubation, aliquots of 100 µL were spread on the surface of Luria Broth (LB, bacteria) solid media or Potato Dextrose Agar (PDA, fungi) and microbial growth was inspected after 24 h of plate incubation at 37 °C. The lowest nanoparticle concentration for which no colony-forming units were detected was considered the minimal bactericidal or fungicidal concentration. Negative controls prepared with sterile culture media and positive controls without nanoparticles were also carried out. Experiments were performed twice using triplicates per experiment.

## 4. Conclusions

In this work, a new class of halogenosilane-functionalized ZnO nanoparticles was successfully developed through a controlled chemical precipitation approach, demonstrating that surface modification represents an efficient strategy to tailor both physicochemical and biological properties of ZnO-based nanomaterials. Structural and morphological analyses confirmed that the incorporation of halogenosilanes leads to significant particle size reduction and improved dispersion, with bromopropylsilane-modified ZnO (ZnO_b) exhibiting the smallest particle size and most uniform morphology among the investigated nanomaterials.

Biological evaluation revealed a clear enhancement of anticancer activity for all halogenosilane-modified ZnO nanoparticles compared to unmodified ZnO, which can be attributed to improved cellular uptake and increased intracellular ROS generation. Interestingly, the relationship between cytotoxicity, apoptosis and ROS production suggests that multiple and potentially competing cell death pathways are involved, depending on nanoparticle surface characteristics. In particular, while unmodified ZnO appears to favor apoptosis-related mechanisms, halogenosilane-modified systems, especially ZnO_b, may induce cell death through more complex ROS-driven processes.

Additional morphological evidence supporting these findings by SEM analysis of treated PC3 cells revealed distinct alterations in cell structure consistent with apoptosis and impaired cellular functionality. These observations reinforce the importance of nanoparticle–cell interactions in determining biological outcomes and further highlight the role of surface functionalization in modulating such interactions.

Overall, the present study demonstrates that halogenosilane functionalization enables modulation of the biological profile of ZnO nanoparticles, leading to enhanced anticancer activity together with distinct microbiocidal responses depending on the microbial system. These findings support the potential of halogenosilane-modified ZnO NPs as anticancer-oriented nanomaterials in prostate cancer cell models, while further studies on additional cancer cell tumor types are required to establish the broader relevance of this biological response.

## Figures and Tables

**Table 1:** Lattice parameters obtained from XRD analyses of the unmodified and halogenosilane-modified ZnO NPs.

Crystallographic Data	ZnO	ZnO_c	ZnO_b	ZnO_i
Crystallinity Degree (%)	59.16	53.10	52.86	49.75
ICDD PDF4+	04-007-1614	04-006-9717	04-007-1614	04-006-9717
Phase	Zincite	Zincite	Zincite	Zincite
Crystallization group	P63mc	P63mc	P63mc	P63mc
Compositional percentage (%)	100	100	100	100
*a* [Å]	3.25244	3.25418	3.254538	3.253683
*b* [Å]	3.25244	3.25418	3.254538	3.253683
*c* [Å]	5.21007	5.21225	5.212538	5.211145
*alpha* [°]	90	90	90	90
*beta* [°]	90	90	90	90
*gamma* [°]	120	120	120	120
Volume [Å^3^]	47.73029	47.80137	47.81439	47.77649
Crystallite size (A)	20.970	7.388	6.919	6.998
Crystal System	Hexagonal	Hexagonal	Hexagonal	Hexagonal
R expected	6.3157	6.14177	6.11882	6.26389
R profile	4.75272	5.06297	5.21976	5.10729
Weighted R profile	6.30025	6.40155	6.64908	6.660314
Goodness of fit	0.99511	1.08638	1.18083	1.13154

**Table 2:** Descriptive statistics of TEM-derived particle size distributions for unmodified and halogenosilane-modified ZnO nanoparticles.

Sample	N	Mean ± SD (nm)	CV (%)	Min–Max (nm)	Skewness	PDI
**ZnO**	100	30.77 ± 7.41	24.07	19.35–51.87	0.88	0.058
**ZnO_c**	100	14.63 ± 6.25	42.75	6.38–39.13	1.80	0.183
**ZnO_b**	100	8.31 ± 2.08	24.97	4.48–12.97	0.25	0.062
**ZnO_i**	100	11.60 ± 4.45	38.39	4.82–27.88	1.22	0.147

**Table 3:** The cytotoxic activity of unmodified ZnO, ZnO_c, ZnO_b, and ZnO_i against the human cancer cells PC3 and 22Rv1, the normal prostate cells RWPE and the human dermal fibroblasts (HDF). Results (mean ± SD) were expressed as the IC_50_ values found upon 24 h incubation.

Sample	IC_50_ (µg/mL)			
	PC3	22Rv1	RWPE	HDF
ZnO	66.0 ± 22	>100	88.7 ± 29	>100
ZnO_c	33.9 ± 17	32.1 ± 5.3	39.9 ± 12	37.1 ± 15
ZnO_b	19.8 ± 6.7	22.3 ± 4.4	27.9 ± 12	25.3 ± 5.0
ZnO_i	35.5 ± 14	35.3 ± 7.3	41.3 ± 14	38.8 ± 14

**Table 4:** Minimal bactericidal or fungicidal concentration (MBC/MFC) of the ZnO and halogenosilane-modified ZnO NPs. The maximal concentration tested was 1500 µg/mL. Results are the means of duplicate experiments performed in triplicate.

NPs	*P. aeruginosa*	*E. coli*	*B. contaminans*	*S. aureus*	*C. albicans*	*C. glabrata*
ZnO	>1500	1500	>1500	1500	>1500	>1500
ZnO_c	>1500	1250	>1500	1250	>1500	>1500
ZnO_b	>1500	1250	>1500	1500	>1500	>1500
ZnO_i	>1500	1250	>1500	1250	>1500	>1500

## Data Availability

The original contributions presented in this study are included in the article. Further inquiries can be directed to the corresponding authors.
